# Using survey data to estimate the impact of the omicron variant on vaccine efficacy against COVID-19 infection

**DOI:** 10.1038/s41598-023-27951-3

**Published:** 2023-01-17

**Authors:** Jesús Rufino, Carlos Baquero, Davide Frey, Christin A. Glorioso, Antonio Ortega, Nina Reščič, Julian Charles Roberts, Rosa E. Lillo, Raquel Menezes, Jaya Prakash Champati, Antonio Fernández Anta

**Affiliations:** 1 CoronaSurveys Team, Madrid, Spain; 2 IMDEA Networks Institute, Av. Mar Mediterráneo 22, 28918 Leganés, Madrid Spain; 3 University of Porto & INESC TEC, Porto, Portugal; 4 Univ Rennes, IRISA, CNRS, Inria, 35042 Rennes, France; 5 Academics for the Future of Science, Inc. & University of California San Francisco, San Francisco, USA; 6 University of Southern California, Los Angeles, USA; 7 Jožef Stefan Institute, Department of Intelligent Systems, Ljubljana, Slovenia; 8 Skyhaven Media, Liverpool, UK; 9 University of Carlos III de Madrid, Getafe, Madrid, Spain; 10 Centre of Mathematics of University of Minho, Braga, Portugal

**Keywords:** Vaccines, Viral infection, Computer science

## Abstract

Symptoms-based detection of SARS-CoV-2 infection is not a substitute for precise diagnostic tests but can provide insight into the likely level of infection in a given population. This study uses symptoms data collected in the Global COVID-19 Trends and Impact Surveys (UMD Global CTIS), and data on variants sequencing from GISAID. This work, conducted in January of 2022 during the emergence of the Omicron variant (subvariant BA.1), aims to improve the quality of infection detection from the available symptoms and to use the resulting estimates of infection levels to assess the changes in vaccine efficacy during a change of dominant variant; from the Delta dominant to the Omicron dominant period. Our approach produced a new symptoms-based classifier, Random Forest, that was compared to a ground-truth subset of cases with known diagnostic test status. This classifier was compared with other competing classifiers and shown to exhibit an increased performance with respect to the ground-truth data. Using the Random Forest classifier, and knowing the vaccination status of the subjects, we then proceeded to analyse the evolution of vaccine efficacy towards infection during different periods, geographies and dominant variants. In South Africa, where the first significant wave of Omicron occurred, a significant reduction of vaccine efficacy is observed from August-September 2021 to December 2021. For instance, the efficacy drops from 0.81 to 0.30 for those vaccinated with 2 doses (of Pfizer/BioNTech), and from 0.51 to 0.09 for those vaccinated with one dose (of Pfizer/BioNTech or Johnson & Johnson). We also extended the study to other countries in which Omicron has been detected, comparing the situation in October 2021 (before Omicron) with that of December 2021. While the reduction measured is smaller than in South Africa, we still found, for instance, an average drop in vaccine efficacy from 0.53 to 0.45 among those vaccinated with two doses. Moreover, we found a significant negative (Pearson) correlation of around − 0.6 between the measured prevalence of Omicron in several countries and the vaccine efficacy in those same countries. This prediction, in January of 2022, of the decreased vaccine efficacy towards Omicron is in line with the subsequent increase of Omicron infections in the first half of 2022.

## Introduction

In early 2022, when this study was conducted, it was clear that the Omicron (B.1.1.529) variant of SARS-CoV-2 (and in particular the subvariant BA.1) was showing an expressive increase since its initial classification in November 2021^1^. In South Africa it appeared to have out-competed the Delta variant^2^ and had rapidly spread into Europe and other regions. Preliminary observations also indicated that it might spread faster and might have higher immune evasiveness than previous variants^3–5^. While vaccination still provided a level of protection against a serious disease^6^, initial results^4,7–9^ pointed towards a reduced level of protection against infection, especially from 15 weeks post the second dose^10^, and it was likely that the number of breakthrough infections (i.e., infections among vaccinated people) would rise with the spread of Omicron^4^. Some of the preliminary models^11^ showed that high transmissibility in combination with high immune evasiveness could lead to a concerning health system overload^12^.

Since the spring of 2020 and until June 2022, the University of Maryland in collaboration with Facebook has collected extensive survey data on self-reported symptoms, infection, testing, behavior and, more recently, vaccination status (UMD Global CTIS)^13,14^. In mid December 2021, researchers used data from this survey concerning the Gauteng province in South Africa to define different combinations of symptoms that are associated with COVID-19 infection, and combined those with self-reported vaccination status to compare vaccine efficacy changes from a Delta dominant period to a subsequent Omicron dominant period^15^. (They used the term *efficacy*, while possibly *effectiveness* is more appropriate^16^). Their findings showed a measurable drop of efficacy towards infection for those vaccinated with two doses. Hansen et al.^17^ published in late December 2021 a study with Danish data from November and December 2021 to report vaccination effectiveness against Omicron (BA.1) with the BNT162b2 (Pfizer/BioNTech) and mRNA-1273 (Moderna) vaccines. They observed that this effectiveness is significantly lower than against Delta infection. As far as we know, these were the only studies available measuring the drop in vaccine effectiveness under Omicron when the present study was completed.

This work, conducted in January of 2022 during the emergence of the Omicron variant (subvariant BA.1), aims to improve the quality of infection detection beyond simple combinations of symptoms, and to use the resulting estimates of infection levels to assess the changes in vaccine efficacy (we use *efficacy* in this paper for consistency with^15^) during a change of dominant variant; from the Delta dominant to the Omicron dominant period. In particular, we use self-reported confirmation of COVID-19 infection, from millions of UMD Global CTIS survey responses, to derive an improved proxy for COVID-19 active cases (using a Random Forest classifier) that tracks more closely the evolution of confirmed cases. Then, we use this improved proxy for analysing prevalence and vaccine efficacy changes in South Africa as a whole, and in its Gauteng province (where the first significant wave of Omicron occurred), among those unvaccinated, partially vaccinated, and fully vaccinated. In South Africa, we observe a significant reduction of vaccine efficacy from August-September 2021 (Delta) to December 2021 (Omicron BA.1). For instance, the efficacy drops from 0.81 to 0.30 for those vaccinated with 2 doses (of Pfizer/BioNTech), and from 0.51 to 0.09 for those vaccinated with one dose (of Pfizer/BioNTech or Johnson & Johnson). We also extended the study to other countries in which Omicron has been detected, comparing the situation in October 2021 (before Omicron) with that of December 2021. While the reduction measured is smaller than in South Africa, we still found, for instance, an average drop in vaccine efficacy from 0.53 to 0.45 among those vaccinated with two doses. Moreover, we found a significant negative (Pearson) correlation of around − 0.6 between the measured prevalence of Omicron in several countries and the vaccine efficacy in those same countries.

This prediction, in January of 2022, of the decreased vaccine efficacy towards Omicron is in line with the sharp increase of Omicron infections in the first half of 2022. It is also consistent with the subsequent literature that has evaluated the efficacy or effectiveness of vaccines against Omicron infection and more severe outcomes conducted in several countries. As mentioned above, Hansen et al.^17^ use Danish data from November and December 2021 to report vaccination effectiveness against Omicron (BA.1). They report effectiveness of 55.2% and 36.7% against Omicron with the BNT162b2 and mRNA-1273 vaccines, respectively, in the first month after primary vaccination and declines rapidly afterwards. (The effectiveness is re-established (54.6%) with a booster shot of the BNT162b2 vaccine.) They also observe that these effectiveness are significantly lower than against Delta infection, which are 86.7% and 88.2% respectively. Andrews et al.^18^ used data from England between November 2021 and January 2022 to study vaccine effectiveness against symptomatic infection with Delta (B.1.617.2) and Omicron (BA.1). They consider three vaccines, BNT162b2 (Pfizer/BioNTech), ChAdOx1 nCoV-19 (Oxford/AstraZeneca), and mRNA-1273 (Moderna). They found that vaccine effectiveness against symptomatic disease was higher for the Delta variant than for the Omicron variant. They found that two doses of ChAdOx1 nCoV-19 provided almost no protection against symptomatic infection with Omicron from 20 to 24 weeks after the second dose. With two BNT162b2 doses, vaccine effectiveness was 65.5% 4 weeks after the second dose, dropping to 8.8% after 25 weeks. Vaccine effectiveness of two doses of mRNA-1273 also reduced from 75.1% after 2 to 4 weeks to 14.9% after 25 or more weeks. While a BNT162b2 or mRNA-1273 booster increased protection, it also waned with time. This study was followed by another one comparing subvariants BA.1 and BA.2 of Omicron^19^. Buchan et al.^20^ use data from Ontario, Canada, in December 2021 to find out that vaccine effectiveness is higher with Delta than with Omicron (BA.1). For instance, 7–59 days after a second dose, effectiveness against symptomatic infection was estimated to be 89% for Delta and only 36% for Omicron. After a third dose, effectiveness against symptomatic infection increased to 97% an 61%, respectively, and was more than 95% against severe outcomes for both variants. Kodera et al.^21^ estimate the effectiveness of vaccination against Delta (September to November 2021) and Omicron (January 2022) in Japan, and how it decreases with time (the waning immunity). They found that the effectiveness of vaccination for the Delta variant was 93% within one month after the second shot. From the reported data of 25,187 positive cases with confirmed Omicron variant in Tokyo in January 2022, the effectiveness of vaccination against Omicron after the second dose is 65% of that of the Delta variant. The literature review done by Chenchula et al.^22^ confirms that boosters increase protection against infection with Omicron. Other new subvariants of Omicron also show high vaccine and antibodies escape^23^.

A difference can also be observed in the effectiveness against hospitalization, although more moderate. In South Africa, Collie et al.^24^ observed a drop in vaccine effectiveness against hospitalization with two doses of BNT162b2 (Pfizer/BioNTech) from 93% in September and October 2021 (Delta) down to 70% in the period November 15 to December 7, 2021 (Omicron). Lauring et al.^25^ evaluate effectiveness of mRNA vaccines. They found that they are highly effective preventing COVID-19 associated hospital admissions against Delta: 85% with two doses and 94% with three doses, while the effectiveness decreases against Omicron: 65% with two doses and 86% with three doses. The effectiveness against in-hospital mortality is also higher against Delta (12.2%) than Omicron (7.1%). Stowe et al.^26^ use data from England to compare vaccine effectiveness against hospitalization with Delta and Omicron. They observe that, while effectiveness is lower and waning is faster for Omicron, this is partially due to incidental cases (hospitalizations not caused by COVID-19), and the difference decreases when only severe respiratory hospitalizations are considered.

As described, these studies find a reduction in the vaccine effectiveness against Omicron versus Delta. Most of them use data obtained from clinical tests, which is time and effort consuming. The use of surveys in^15^ and in this study is a fast and cheap alternative to get estimates of this decrease in vaccine efficacy and effectiveness.

## Methods

### Self-reported survey data

We use the responses to the UMD Global CTIS, which collected more than 100,000 responses daily across the world (except in the US, where the survey is run by CMU^27^). A ‘response’ refers to the answers given by a participant to the questions in the survey. Data covers the period between June and December 2021, and data analysis was conducted in January 2022 during the rise of Omicron in Europe. Note that our choice of the chosen period is guided by the fact that this is the period South Africa is most affected by COVID as there was surge in the number of cases due to the Delta variant from mid-June till mid-November followed by a surge, for the first time in the world, due to the Omicron variant.

We have access to the responses collected by agreement with UMD and Facebook (see Section “Ethical declaration”). All the participants in the CTIS have declared to be at least 18 years of age. The questions of the survey can be found in the UMD Global CTIS site^28^. The responses we use only contain four questions whose answer is quantitative (number of days with symptoms, number of symptomatic contacts, number of people staying at the same place, and years of education completed). We only use these questions to filter abnormal responses (see below). The rest of survey questions we use have categorical answers (see Supplements C and D). In this study we used responses to the UMD Global CTIS from the 50 countries from which the largest amount of data is available. From these we selected 24 countries as described below. The total number of survey responses used from these countries is roughly 2.500.000 (see eTable 5).

The first step is curating the responses (removing abnormal responses), as proposed in Alvarez et al. ^29^. We remove responses that declare to have all symptoms or that declare unusual values (greater than 100) in the quantitative questions of the survey (i.e., number of days with symptoms, number of symptomatic contacts, number of people staying at the same place, years of education completed). After that, the quantitative columns are removed from the data set.

In order to classify the responses as positive or negative, several criteria have been proposed in the literature. In particular, we consider the following symptom-based COVID-like illness classifiers (see Supplement C for the list of symptoms collected in the survey):

- UMD CLI^29,30^: A response is considered to be positive if it declares fever (symptom B1_1), along with cough (symptom B1_2), or shortness of breath/difficulty breathing (symptom B1_3). Otherwise, it is negative.
- Stringent CLI^15^: A response is positive if it declares anosmia (symptom B1_10), combined with fever (B1_1), muscle pain (B1_6), or cough (B1_2). Otherwise, it is negative.
- Classic CLI^15^: A response is positive if it declares cough (B1_2), combined with fever (B1_1), muscle pain (B1_6), or anosmia (B1_10). Otherwise, it is negative.
- Broad CLI^15^: A response is positive if it declares muscle pain (B1_6), combined with fever (B1_1), cough (B1_2), or anosmia (B1_10). Otherwise, it is negative.

These methods for classifying cases as positive or negative have two main limitations. First, they do not take into account diagnostic uncertainty, e.g., the same set of symptoms might be associated with some other condition. Second, these criteria are not adaptive to possible changes in the symptoms experienced as conditions change, e.g., as vaccination rates increase or new virus variants emerge. Thus, in this work, we introduce a new machine-learning-based classifier (described in Supplement B). We build a *ground-truth set* with the responses to the survey that report having been tested in the latest 14 days and know the outcome of the test. The responses in the ground-truth set are used to train a model, which is then used to determine the status of users outside that set (users who do not report test information). We use the random-forest technique to design this classifier and the corresponding results are labeled Random Forest in what follows.

We refer to the values obtained with each of these five classifiers (namely, Random Forest, UMD CLI, Stringent CLI, Classic CLI, and Broad CLI) as *proxy estimates* (or proxy for short). We compare each proxy estimate with the estimate of active cases obtained from the official number of cases as described by Alvarez et al.^29^, where each new case is assumed to remain active for 10 days. These last estimates are called Confirmed.

In the Supplementary Information (Section B.3), the performance of the different proxies are compared. When several ground-truth sets are divided into training and testing subsets (70–30% split), Random Forest almost always shows the highest accuracy, sensitivity / recall, specificity, and F-score (see eTable 9). Similarly, Random Forest almost always shows the largest correlation with Confirmed in a set of 21 countries (see eTable 10).

### Prevalence and efficacy estimation

The prevalence of COVID-19 estimated by a given classifier is the ratio between the number of positive cases over the total number of responses. Then, we consider four subsets of responses:

- Unvaccinated: Participants that respond negatively to the question “V1: Have you had a COVID-19 vaccination?”
- Vaccinated: Participants that respond positively to Question V1.
- Vaccinated with 1 dose: Participants that respond positively to Question V1 and declare having received 1 dose in Question “V2: How many COVID-19 vaccinations have you received?”
- Vaccinated with 2 doses: Participants that respond positively to Question V1 and declare having received 2 doses in Question V2.

Unfortunately, from the questions in the UMD Global CTIS dataset it is not possible to know whether those with one dose are fully vaccinated, i.e., they have received a one-dose vaccine, or they simply received only the first dose of a two-dose vaccination. Similarly, it is not possible to know whether a survey respondent received a booster shot.

For each of these subsets, the prevalence of COVID-19 is computed as the fraction of responses classified as positive among the responses that report a given vaccination status. To be more precise, let us consider a set *R* of responses, and its subsets *R*(*A*) of responses considered to be positive by proxy $A \in \{ \textsf {Random, Forest}, \textsf {UMD  CLI}, \textsf {Stringent  CLI}, \textsf {Classic  CLI}, \textsf {Broad  CLI} \}$. Then, the prevalence *P*(*L*, *A*) among vaccination level $L \in \{\textsf {Unvaccinated}, \textsf {Vaccinated}, \textsf {Vaccinated  with  1   dose}, \textsf {Vaccinated   with   2   doses}\}$ obtained with proxy *A* is defined as

$$\begin{aligned} P(L,A)=\frac{|R(A) \cap L|}{|R|}. \end{aligned}$$

For each proxy we also estimate the *vaccine efficacy* (*VE*) against illness as in^15^, based on the estimates of prevalence among unvaccinated and vaccinated. The vaccine efficacy $$ VE (L,A)$$ of vaccination level *L* with proxy *A* is defined as

$$\begin{aligned} VE (L,A)=1-\frac{P(L,A)}{P(\textsf {Unvaccinated},A)}. \end{aligned}$$

The confidence intervals of this metric are obtained using the Katz-log Method^31^. Since we have three subsets of vaccinated participants, we compute the vaccine efficacy for the vaccination levels Vaccinated, Vaccinated with 1 dose, and Vaccinated with 2 doses.

### Countries and time periods

#### South Africa

The main objective of this work is to evaluate the change in vaccine efficacy due to the Omicron variant. To this end, we evaluate the decrease in vaccine efficacy in South Africa and the Gauteng province from mid-June 2021 until the end of 2021. Our motivation for choosing South Africa as the main country of this study is that it was the first country in which the Omicron variant was detected, and had its highest prevalence in the period of this study. Further, the Gauteng province was the region of origin of this variant. In the period considered, South Africa (and Gauteng in particular) were strongly affected by COVID, as there was surge in the number of cases due to the Delta variant from mid-June till mid-November, followed by a surge due to the Omicron variant in December.

Moreover, to ensure that we have sufficient data for our estimates, we concentrate on three relevant time periods, each lasting about a month, where more data is available. During two of these time periods the Delta variant is dominant: (i) June 18 to July 18, 2021, the period considered in^15^ with low vaccination level (see eFigure 1), and (ii) August 9 to September 6, 2021; while in the last time period, December 1st to 31st, 2021, Omicron is dominant (see eTable 1; the information on variant presence is obtained from^32^, which extracts it from^33^ via^2^). Period (i) is included for completeness and comparison with^15^, but as can be seen in eTables  1 and 2, the vaccination level in that period is low and the relative presence of Delta is lower than in Period (ii). In any case, the vaccine efficacies observed in both Delta periods are comparable (see Table 1).

**Table 1:** Vaccine efficacy in South Africa and the Gauteng province, calculated for three time periods: June 18th to July 18th (Jun–Jul), August 9th to September 6th (Aug–Sep), and December 1st to 31st (Dec).

Method	Jun–Jul efficacy [95% CI]	Aug–Sep efficacy [95% CI]	Dec efficacy [95% CI]
South Africa			
Vaccinated			
Random Forest	0.54 [0.48, 0.59]	0.62 [0.58, 0.65]	0.24 [0.17, 0.30]
UMD CLI	0.60 [0.53, 0.66]	0.66 [0.61, 0.70]	0.46 [0.39, 0.51]
Stringent CLI	0.69 [0.63, 0.74]	0.70 [0.66, 0.73]	0.48 [0.40, 0.55]
Classic CLI	0.55 [0.50, 0.59]	0.56 [0.52, 0.59]	0.38 [0.33, 0.43]
Broad CLI	0.50 [0.44, 0.54]	0.49 [0.44, 0.52]	0.36 [0.30, 0.41]
Vaccinated with one dose			
Random Forest	0.50 [0.44, 0.56]	0.51 [0.46, 0.55]	0.09 [0.00, 0.18]
UMD CLI	0.61 [0.54, 0.68]	0.56 [0.50, 0.62]	0.21 [0.09, 0.31]
Stringent CLI	0.67 [0.61, 0.73]	0.60 [0.54, 0.65]	0.23 [0.07, 0.36]
Classic CLI	0.53 [0.47, 0.57]	0.47 [0.42, 0.51]	0.21 [0.13, 0.28]
Broad CLI	0.46 [0.40, 0.52]	0.39 [0.34, 0.44]	0.18 [0.09, 0.26]
Vaccinated with two doses			
Random Forest	0.76 [0.64, 0.84]	0.81 [0.78, 0.84]	0.30 [0.23, 0.36]
UMD CLI	0.75 [0.57, 0.86]	0.85 [0.79, 0.88]	0.56 [0.50, 0.61]
Stringent CLI	0.82 [0.66, 0.90]	0.88 [0.84, 0.91]	0.59 [0.51, 0.65]
Classic CLI	0.77 [0.66, 0.84]	0.71 [0.67, 0.75]	0.45 [0.40, 0.49]
Broad CLI	0.75 [0.63, 0.83]	0.66 [0.61, 0.71]	0.43 [0.37, 0.48]
Gauteng			
Vaccinated			
Random Forest	0.43 [0.33, 0.51]	0.62 [0.54, 0.69]	0.30 [0.18, 0.40]
UMD CLI	0.58 [0.44, 0.68]	0.63 [0.51, 0.73]	0.52 [0.41, 0.61]
Stringent CLI	0.64 [0.53, 0.72]	0.70 [0.61, 0.78]	0.57 [0.43, 0.67]
Classic CLI	0.50 [0.42, 0.58]	0.51 [0.42, 0.59]	0.48 [0.39, 0.55]
Broad CLI	0.49 [0.39, 0.57]	0.41 [0.31, 0.50]	0.45 [0.35, 0.53]
Vaccinated with one dose			
Random Forest	0.40 [0.28, 0.49]	0.54 [0.44, 0.63]	0.14 [0.00, 0.30]
UMD CLI	0.60 [0.46, 0.71]	0.58 [0.42, 0.70]	0.38 [0.18, 0.53]
Stringent CLI	0.62 [0.49, 0.71]	0.61 [0.47, 0.71]	0.39 [0.13, 0.57]
Classic CLI	0.47 [0.37, 0.56]	0.47 [0.36, 0.56]	0.35 [0.20, 0.46]
Broad CLI	0.44 [0.33, 0.53]	0.34 [0.20, 0.45]	0.29 [0.14, 0.42]
Vaccinated with two doses			
Random Forest	0.62 [0.36, 0.78]	0.77 [0.67, 0.85]	0.36 [0.24, 0.46]
UMD CLI	0.69 [0.27, 0.87]	0.73 [0.54, 0.84]	0.57 [0.45, 0.66]
Stringent CLI	0.85 [0.55, 0.95]	0.88 [0.76, 0.94]	0.65 [0.51, 0.74]
Classic CLI	0.79 [0.59, 0.90]	0.58 [0.44, 0.68]	0.53 [0.44, 0.60]
Broad CLI	0.80 [0.59, 0.91]	0.54 [0.39, 0.65]	0.50 [0.41, 0.58]

#### World

Beyond South Africa, we study the 50 countries for which the UMD Global CTIS has the largest amount of data. For all of them we compute the vaccine efficacy with proxy Random Forest in the month of October 2021 (in which Omicron was still not present) and in the month of December 2021 (in which Omicron was present). A computed efficacy value is only considered if (i) it is non-negative, (ii) both prevalences $P(L,\textsf {Random  Forest})$ and $P(\textsf {Unvaccinated},  \textsf {Random Forest})$ are at least 0.01, and (iii) the number of samples used to compute them is at least 1000. We only consider further those countries for which these three conditions hold for the efficacy value in December of at least one among the vaccination status cases we consider.

We have observed that the information on prevalence of Omicron becomes available^32^ with a significant delay. Hence, most countries do not report relevant presence of Omicron until the second half of December 2021. For that reason, we consider the prevalence of Omicron reported from December 15th, 2021 to January 7th, 2022. Furthermore, among the countries mentioned above, in order to have a reasonable estimate of the prevalence of the Omicron variant, we consider only countries whose data is based on sequencing at least 30 virus samples. We say that these are the countries with *presence of Omicron*. For all countries with presence of Omicron, we compare the estimated vaccination efficacy using Random Forest among all three vaccination groups and for both periods. For this, we adopt simple statistical methods, such as correlation analysis.

### Ethical declaration

The Ethics Board (IRB) of IMDEA Networks Institute gave ethical approval for this work on 2021/07/05. IMDEA Networks has signed Data Use Agreements with Facebook, Carnegie Mellon University (CMU) and the University of Maryland (UMD) to access their data, specifically UMD project 1587016-3 entitled C-SPEC: Symptom Survey: COVID-19 and CMU project STUDY2020_00000162 entitled ILI Community-Surveillance Study. The data used in this study was collected by the University of Maryland through The University of Maryland Social Data Science Center Global COVID-19 Trends and Impact Survey in partnership with Facebook. Informed consent has been obtained from all participants in this survey by this institution (see^28^). All the methods in this study have been carried out in accordance with relevant of ethics and privacy guidelines and regulations.

## Results

### Prevalence and vaccination efficacy in South Africa

**Figure 1:**
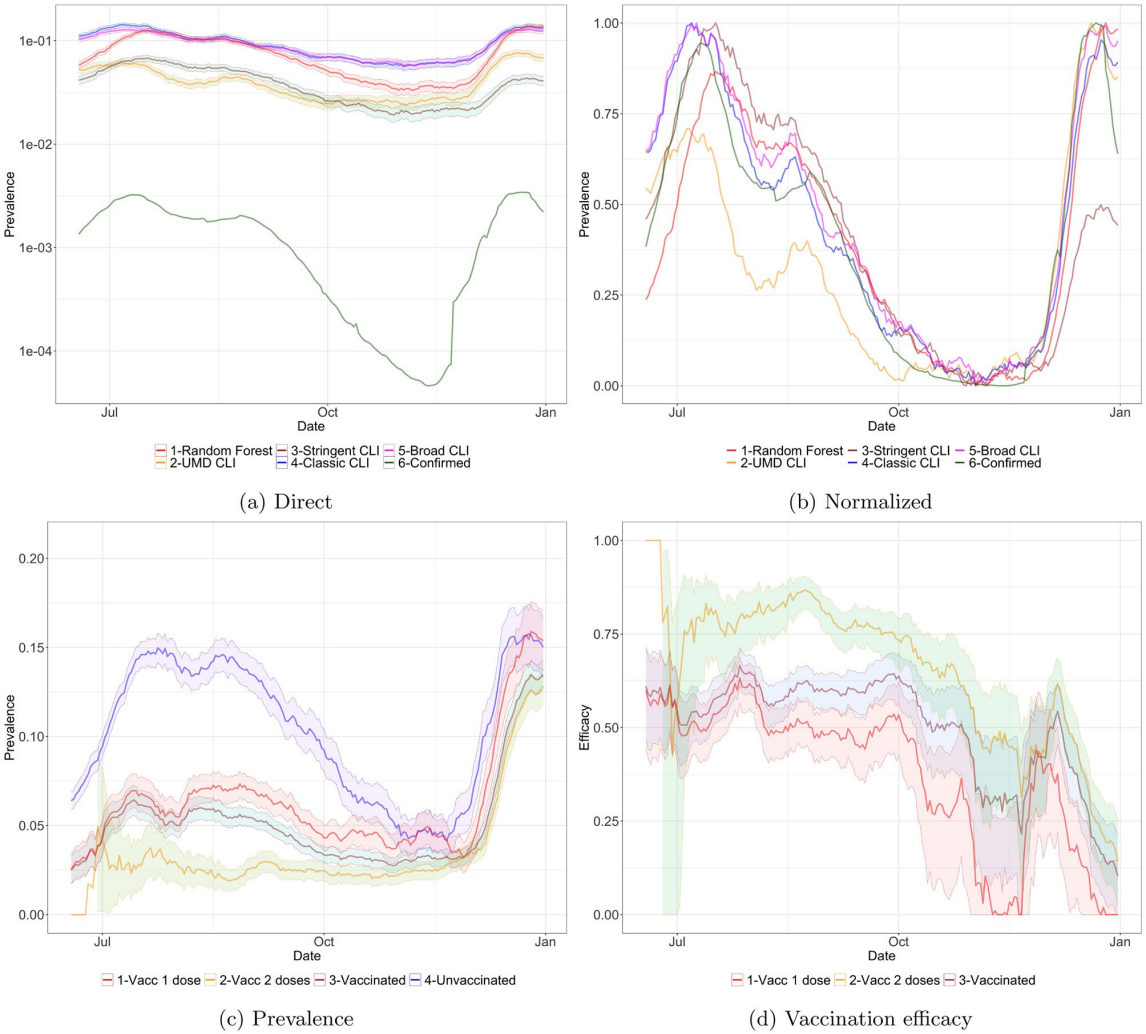
(**a**, **b**) Prevalence in South Africa obtained with the different proxies, smoothed with a rolling average of 14 days from June 18th to December 31st, 2021. In plot (**a**) we have the actual ratio (note that the y axis is in logarithmic scale). In plot (**b**) all curves are normalized so the smallest value is 0 and the largest value is 1. (**c**) Prevalence and (**d**) vaccination efficacy in South Africa among people with different levels of vaccination, estimated with Random Forest.

Figure 1a and b show the prevalence of COVID-19 in South Africa in the period June 18th to December 31st, 2021, with the different proxies. The direct approach of Fig. 1a shows a gap between the estimate Confirmed derived from the official number of cases and the other proxies. This gap can be explained in part by under-detection in the official number of cases. (In South Africa the test-positivity rate -TPR- is above 15%, as seen in eTable 10, while the WHO recommends to have a maximum TPR of 5% to track the number of cases appropriately^34^). More generally (in South Africa and elsewhere) symptom-based proxies can overestimate the number of cases when respondents report symptoms that are consistent with COVID-19 but are produced by some other condition. Figure 1b shows that if each curve is independently normalized to the unit scale, all proxies closely track the evolution of the official number of cases Confirmed.

The Supplementary Information shows the proportion of CTIS responses with each vaccinated status, and the fraction of the population that received each type of vaccine from the two used in South Africa, Johnson & Johnson and Pfizer/BioNTech (see eFigure 1 and eTable 10). As can be seen even on January 2022 there were more than 20% of the CTIS responses that reported no vaccination, so the unvaccinated sample is large during the whole period of interest. In eFigure 2a–d we show the COVID-19 prevalence in South Africa depending on the vaccination status with the different proxies. Focusing on the unvaccinated curve, we can observe that the UMD CLI and Stringent CLI proxies show a low infection prevalence in July–September 2021 and December 2021 when compared with the other proxies. On the other hand, Classic CLI and Broad CLI show a high prevalence in the period October–November 2021, when the official data was showing that the number of cases was very low, possibly because of existing symptoms in the population not related to COVID-19. We hence focus in the Random Forest proxy which seems to be more reliable.

Figure 1c shows the prevalence in South Africa across all reported vaccination states with the Random Forest proxy. We can observe that the magnitude of the two waves (August–September and December 2021) is similar among the Unvaccinated population, while in the vaccinated groups (Vaccinated, Vaccinated with 1 dose and Vaccinated with 2 doses) there is a much higher rate of prevalence in the December 2021 wave. This hints at a decrease of vaccine efficacy towards infection with the introduction of Omicron, as we will show next. We also observe that, as expected, subjects vaccinated with two doses show higher protection that those reporting only one dose (with Vaccinated somewhere in between since it combines both groups). In fact, the prevalence among unvaccinated and vaccinated with one dose are almost the same in December 2021, even though a large fraction was vaccinated with a one-dose vaccine (Johnson & Johnson, see eTable 1).

As for vaccination efficacy, Fig. 1d shows the estimates for South Africa, again with Random Forest. While the data in October–November 2021 has lower quality due to the reduced number of cases in that country, we can clearly observe the reduction of vaccine efficacy, towards infection, when contrasting the August–September 2021 period to the December 2021 period, when Omicron dominates. Observe that vaccination with one dose has zero efficacy at the end of December 2021. Table 1 quantifies the estimated efficacy for the three periods of interest and for the five classifiers, for South Africa and for the Gauteng province. As can be observed, the efficacy with two doses in South Africa drops from August-September to December 2021 by at least 0.2 with most proxies (0.38 with Random Forest), and even more with one dose (0.42 with Random Forest).

### Prevalence and vaccination efficacy in the world

From the 50 countries with the largest amount of data in the CTIS and having *presence of Omicron*, we select those with an acceptable estimated efficacy value (where estimates are accepted if they follow the three rules listed in Section 2.3), resulting in a set of 24 countries. As a reference, in Supplementary Information, Section F, we show the level of vaccination in these countries (eTable 3) and we list the vaccine types delivered in each country (eTable 1). (Vaccination data is obtained from^32,35,36^). Then, Tables 2 and 3, present the estimates of virus prevalence in the same countries in the periods of October and December 2021, and also estimates of vaccination efficacy towards infection.

Both prevalence estimates and the derived efficacy estimates are obtained by the Random Forest classifier and shown with 95% confidence intervals. When data is insufficient to meet the defined selection criteria (c.f. Section 2.3), it is omitted and replaced by “–”. Both tables are presented alphabetically by country name and also share a column depicting the most recent data on Omicron prevalence among all virus samples. While Table 2 focuses on the data from individuals that declared their overall vaccination status (using groups Vaccinated, Unvaccinated), Table 3 makes a more detailed characterization by considering the number of doses declared (groups Vaccinated with 1 dose, Vaccinated with 2 doses, Unvaccinated). We also observe that there is less data on individuals with only one dose, since this is a transient state in the vaccination sequence. The full information on sample sizes can be consulted in eTables 5 and 6.

**Table 2:** Prevalence of Omicron in COVID-19 and vaccination efficacy in the countries with presence of Omicron (as defined in Section “Countries and time periods”).

Country	% Prevalence	Prevalence	Prevalence	Vac efficacy	Vac efficacy
	Omicron	Oct	Dec	Oct	Dec
Argentina	0.83 [0.76, 0.91]	0.02 [0.01, 0.02]	0.03 [0.03, 0.03]	0.48 [0.35,0.58]	0.28 [0.12,0.41]
Belgium	0.32 [0.29, 0.34]	0.02 [0.02, 0.02]	0.05 [0.05, 0.05]	0.53 [0.39,0.64]	0.38 [0.26,0.48]
Brazil	0.58 [0.52, 0.64]	0.03 [0.03, 0.03]	0.03 [0.02, 0.03]	0.43 [0.37,0.49]	0.29 [0.19,0.38]
Colombia	0.35 [0.26, 0.44]	0.03 [0.03, 0.03]	0.03 [0.03, 0.03]	0.55 [0.49,0.61]	0.49 [0.39,0.56]
Denmark	0.47 [0.46, 0.49]	0.01 [0.01, 0.01]	0.05 [0.05, 0.05]	–	0.49 [0.39,0.57]
France	0.26 [0.24, 0.27]	0.01 [0.01, 0.01]	0.03 [0.03, 0.03]	–	0.44 [0.39,0.49]
Germany	0.13 [0.13, 0.14]	0.01 [0.01, 0.01]	0.02 [0.02, 0.02]	–	0.65 [0.62,0.68]
India	0.33 [0.29, 0.38]	0.04 [0.04, 0.04]	0.03 [0.03, 0.03]	0.44 [0.35,0.52]	0.42 [0.28,0.53]
Italy	0.21 [0.19, 0.22]	0.01 [0.01, 0.01]	0.02 [0.02, 0.02]	–	0.61 [0.57,0.65]
Mexico	0.54 [0.49, 0.58]	0.05 [0.05, 0.05]	0.04 [0.04, 0.04]	0.57 [0.54,0.59]	0.51 [0.46,0.55]
Netherlands	0.30 [0.27, 0.33]	0.02 [0.02, 0.02]	0.05 [0.04, 0.05]	0.36 [0.20,0.49]	0.29 [0.18,0.38]
Norway	0.25 [0.15, 0.36]	0.01 [0.01, 0.01]	0.03 [0.02, 0.03]	–	0.35 [0.10,0.52]
Poland	0.03 [0.02, 0.04]	0.03 [0.03, 0.04]	0.07 [0.06, 0.07]	0.50 [0.42,0.56]	0.57 [0.53,0.60]
Portugal	0.23 [0.19, 0.27]	0.01 [0.01, 0.01]	0.03 [0.03, 0.03]	–	0.32 [0.12,0.48]
Romania	0.04 [0.00, 0.08]	0.06 [0.06, 0.06]	0.02 [0.02, 0.02]	0.59 [0.56,0.62]	0.65 [0.57,0.71]
Russia	0.29 [0.22, 0.36]	0.04 [0.04, 0.05]	0.03 [0.02, 0.03]	0.45 [0.39,0.50]	0.43 [0.34,0.51]
Slovakia	0.10 [0.03, 0.17]	0.03 [0.03, 0.03]	0.06 [0.05, 0.06]	0.47 [0.32,0.59]	0.54 [0.46,0.61]
South Africa	0.88 [0.81, 0.96]	0.04 [0.04, 0.04]	0.12 [0.12, 0.13]	0.50 [0.41,0.57]	0.24 [0.17,0.30]
Spain	0.46 [0.43, 0.50]	0.01 [0.01, 0.02]	0.05 [0.05, 0.06]	0.62 [0.50,0.70]	0.26 [0.15,0.36]
Sweden	0.34 [0.32, 0.37]	0.01 [0.00, 0.01]	0.02 [0.02, 0.02]	–	0.48 [0.36,0.57]
Switzerland	0.39 [0.36, 0.41]	0.01 [0.01, 0.01]	0.04 [0.04, 0.04]	–	0.52 [0.43,0.59]
Turkey	0.10 [0.08, 0.11]	0.05 [0.05, 0.06]	0.05 [0.05, 0.05]	0.45 [0.38,0.51]	0.42 [0.33,0.51]
United Kingdom	0.66 [0.65, 0.66]	0.03 [0.03, 0.03]	0.05 [0.04, 0.05]	0.34 [0.22,0.45]	0.20 [0.07,0.31]
Vietnam	0.02 [0.00, 0.06]	0.01 [0.01, 0.01]	0.03 [0.03, 0.03]	–	–

When data is insufficient to meet the defined selection criteria, it is omitted and replaced by “–”.

**Table 3:** Prevalence of Omicron and vaccination efficacy with one and two doses in the countries with presence of Omicron (as defined in Section “Countries and time periods”).

Country	% Prevalence	Vac 1 dose	Vac 1 dose	Vac 2 doses	Vac 2 doses
	Omicron	Efficacy Oct	Efficacy Dec	Efficacy Oct	Efficacy Dec
Argentina	0.83 [0.76, 0.91]	0.03 [0.00, 0.27]	–	0.53 [0.41, 0.62]	0.31 [0.15, 0.43]
Belgium	0.32 [0.29, 0.34]	–	–	0.55 [0.41, 0.65]	0.38 [0.26, 0.48]
Brazil	0.58 [0.52, 0.64]	0.20 [0.11, 0.28]	–	0.50 [0.44, 0.55]	0.33 [0.23, 0.41]
Colombia	0.35 [0.26, 0.44]	0.44 [0.35, 0.53]	0.36 [0.22, 0.47]	0.61 [0.55, 0.67]	0.53 [0.45, 0.61]
Denmark	0.47 [0.46, 0.49]	–	–	–	0.48 [0.38, 0.57]
France	0.26 [0.24, 0.27]	–	0.46 [0.35, 0.55]	–	0.44 [0.39, 0.49]
Germany	0.13 [0.13, 0.14]	–	0.44 [0.34, 0.53]	–	0.66 [0.63, 0.69]
India	0.33 [0.29, 0.38]	0.19 [0.05, 0.31]	0.07 [0.00, 0.26]	0.54 [0.47, 0.61]	0.49 [0.37, 0.58]
Italy	0.21 [0.19, 0.22]	–	0.66 [0.57, 0.72]	–	0.61 [0.56, 0.65]
Mexico	0.54 [0.49, 0.58]	0.36 [0.32, 0.40]	0.22 [0.14, 0.30]	0.66 [0.63, 0.68]	0.56 [0.52, 0.60]
Netherlands	0.30 [0.27, 0.33]	–	0.16 [0.00, 0.33]	0.41 [0.26, 0.53]	0.30 [0.19, 0.39]
Norway	0.25 [0.15, 0.36]	–	–	–	0.35 [0.11, 0.53]
Poland	0.03 [0.02, 0.04]	0.31 [0.13, 0.45]	0.44 [0.34, 0.52]	0.52 [0.45, 0.58]	0.58 [0.55, 0.62]
Portugal	0.23 [0.19, 0.27]	–	0.23 [0.00, 0.44]	–	0.33 [0.13, 0.49]
Romania	0.04 [0.00, 0.08]	0.65 [0.59, 0.70]	0.52 [0.33, 0.65]	0.58 [0.55, 0.61]	0.68 [0.60, 0.74]
Russia	0.29 [0.22, 0.36]	0.55 [0.43, 0.64]	0.30 [0.09, 0.46]	0.44 [0.38, 0.50]	0.46 [0.37, 0.53]
Slovakia	0.10 [0.03, 0.17]	–	–	0.50 [0.35, 0.61]	0.55 [0.47, 0.62]
South Africa	0.88 [0.81, 0.96]	0.29 [0.15, 0.40]	0.09 [0.00, 0.18]	0.64 [0.56, 0.70]	0.30 [0.23, 0.36]
Spain	0.46 [0.43, 0.50]	0.34 [0.09, 0.52]	0.30 [0.15, 0.43]	0.66 [0.55, 0.74]	0.26 [0.14, 0.36]
Sweden	0.34 [0.32, 0.37]	–	–	–	0.48 [0.36, 0.57]
Switzerland	0.39 [0.36, 0.41]	–	–	–	0.51 [0.42, 0.59]
Turkey	0.10 [0.08, 0.11]	–	–	0.49 [0.42, 0.55]	0.44 [0.34, 0.52]
United Kingdom	0.66 [0.65, 0.66]	–	–	0.36 [0.24, 0.46]	0.21 [0.08, 0.32]
Vietnam	0.02 [0.00, 0.06]	–	0.25 [0.00, 0.50]	–	–

When data is insufficient to meet the defined selection criteria, it is omitted and replaced by “–”. The prevalence of Omicron is replicated from Table 2 for easy reference.

Comparing these results with other studies, we observe that the efficacy in Denmark after two doses is 0.48, when Hansen et al.^17^ claimed that in the first month after the second dose the observed effectiveness is 55.20% with BNT162b2 (Pfizer/BioNTech) and 36.70% with mRNA-1273 (Moderna). In United Kingdom we observe an efficacy of 0.21 with two doses, while Andrews et al.^18^ reports effectiveness of BNT162b2 (Pfizer/BioNTech) being 65.50% in the first 4 weeks, dropping to 8.80% after 25 weeks, no effectiveness of ChAdOx1 nCoV-19 (Oxford/AstraZeneca) after 20 weeks, and effectiveness of mRNA-1273 (Moderna) being 75.1% in the first 4 weeks, dropping to 14.9% after 25 weeks.

**Figure 2:**
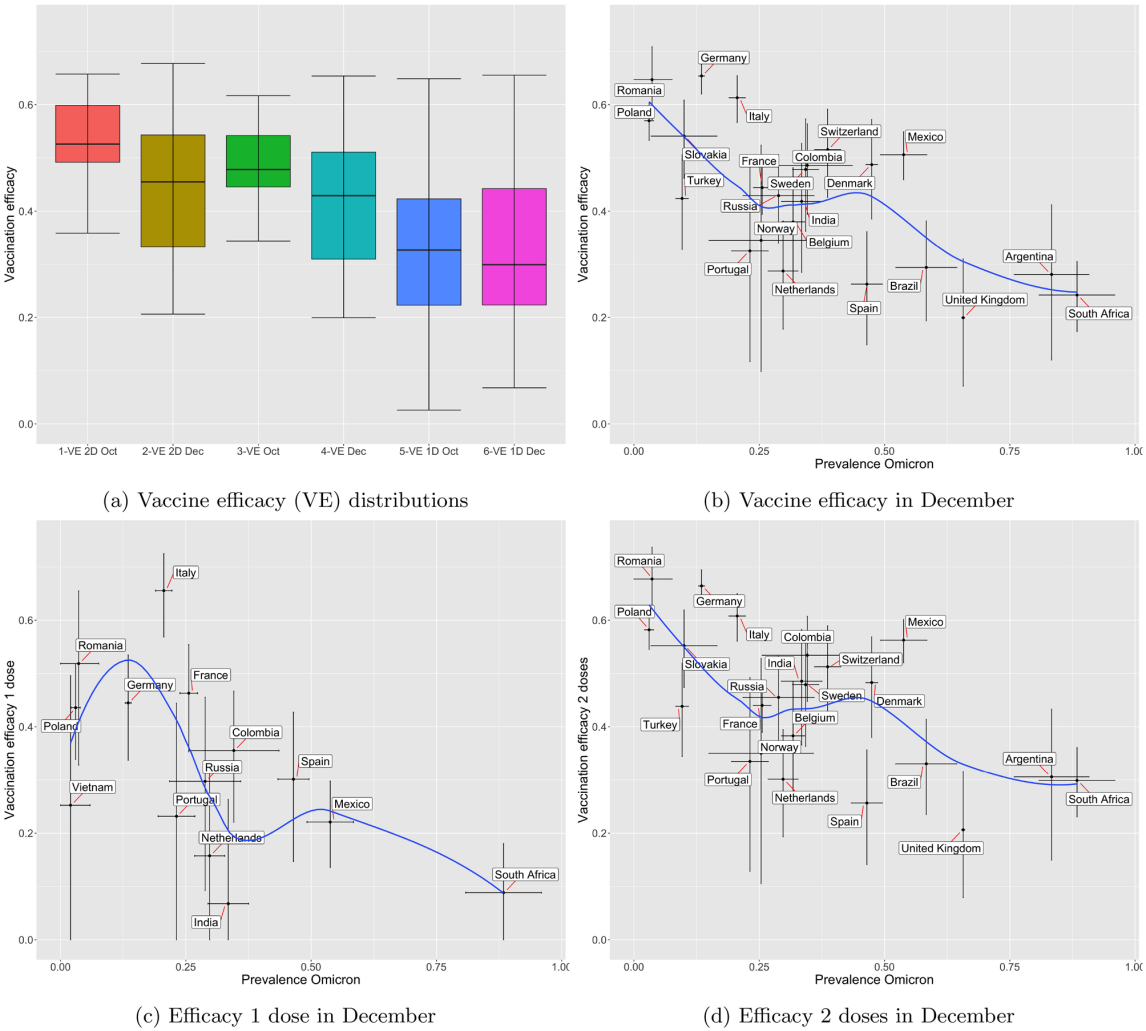
Analysis of vaccine efficacy towards preventing infection: Sub-figure (**a**) shows distributions of efficacy in October and December, for the countries with presence of Omicron (as defined in Section “Countries and time periods”); Sub-figures (**b**–**d**) show vaccination efficacy versus Omicron prevalence in the same set of countries, depending on vaccination status. For each country the 95% confidence intervals of the two values are shown as black lines. The blue line is the Loess curve fitting of the data.

Figure 2a shows three pairs of box plots. Each pair allows comparing vaccine efficacy in October and December 2021 when considering data from the selected countries. eTable 7 presents the average corresponding to each boxplot, with the 95% confidence interval. We observe that although results are inconclusive for Vaccinated with 1 dose, there is a clear decrease of overall efficacy when considering Vaccinated and Vaccinated with 2 doses.

Figure 2b–d allow us to see a clear trend when plotting efficacy against the most recent relative level of Omicron presence in each selected country. For each case, we present a smoothed line (Loess fitting curve, in blue), depicting a clear decreasing trend. eTable 8 presents estimates for the correlation coefficient (using Pearson correlation) together with the corresponding p-value, which confirms its statistical significance for the usual $\alpha =5\%$.

## Discussion

After its surge in South Africa, the Omicron variant increased in prevalence in other countries. In January 2022 it was still unclear if this variant was associated to a milder disease^37^ several studies have raised concerns over the decrease of vaccine efficacy against infection^4,7–9^ and this was predicted and turned out to lead to a wider spread of the virus even in countries with a high vaccination uptake.

Daily participatory symptom surveillance has the potential to offer a new instrument for assessing both global and local trends in health status. While limited in assessing the ground truth, due to the smaller control over the sample design and the need to preserve anonymity, we believe that the vast number of daily survey responses can compensate some of these factors. In this study, we developed a method to adapt and calibrate against the reported SARS-CoV-2 infection status the selection of symptoms, and other covariates from the survey, along different time periods and locations. As compared to methods that only use the presence or absence of symptoms reported by survey respondents^15^, our proposed method was shown to provide a better proxy for assessing the trend in infections, more closely tracking the official reported cases, in particular in those countries that had a strong surveillance and consistent test positivity rates.

Using this improved classifier we complemented earlier results^15^ that used traditional fixed combinations of symptoms, and updated the analysis for South Africa showing the observed decrease in vaccine efficacy when contrasting a Delta-dominated period (August–September 2021) with the following Omicron-dominated period (December 2021). We confirmed the presence of a measurable drop in vaccine efficacy from 0.62 (with 95% confidence interval [0.58, 0.65]) in the Delta period to 0.24 (95% CI [0.17, 0.30]) in the Omicron period in the whole country (0.62 [0.54, 0.69] to 0.30 [0.18, 0.40] in the Gauteng province). In addition, we confirmed that having two doses of vaccine confers better protection than one dose, both in Delta (0.81 [0.78, 0.84] versus 0.51 [0.46, 0.55]) and Omicron (0.30[0.23, 0.36] versus 0.09[0.00, 0.18]) dominated periods. However, we have no data on the status of respondents with regard to a possible booster dose.

By January 7th, 2022, there was a small number of candidate countries exhibiting both a high prevalence of Omicron and a high level of sequencing data supporting it. Nevertheless, we extend our analysis to these countries and show the observed changes in efficacy when comparing the months of October (pre-Omicron) with December (with partial presence of Omicron). Although these results could be re-tuned once the level of Omicron became more dominant in many countries, we have already observed in January 2022, when the data was processed and analysed, a significant level of correlation of around and beyond − 0.62 between vaccine efficacy (with either one or two doses) and the prevalence of Omicron. We must also make it clear that our results show a reduction in efficacy in terms of protection against infection, but this does not imply a similar reduction of vaccine efficacy in protection against serious disease, hospitalization and death. In fact, Collie et al.^24^ observed a moderate drop in vaccine effectiveness against hospitalization in South Africa with two doses of Pfizer/BioNTech, from 93% in September and October 2021 (Delta) down to 70% in the period November 15 to December 7, 2021 (Omicron). This moderate decrease in effectiveness against hospitalization was also observed in other studies^25,26^. The wide spread of Omicron infections during the first half of 2022 that was observed after this study was performed, and the moderate impact in hospitalization and fatality figures in vaccinated countries gives support to these observations^36^.

There are several assumptions that frame our analysis. We assume that UMD Global CTIS answers provide a sample of the population that is interchangeable among the Delta and Omicron dominated periods. We are aware that the responses of the UMD Global CTIS are biased. For starters, only Facebook users can fill the survey, who in some countries represent a small fraction of the population and in most countries are distributed unevenly among age groups. However, we have observed other selection biases in CTIS. For example, in eTable 1 of the Supplementary Information can be seen that in South Africa the percentage of vaccinated persons responding the survey is higher than the official percentage of the population vaccinated. However, we do not believe these biases influence substantially the results of this study.

Additionally, we did not take into account possible effects from waning immunity and vaccine boost shots. However, within the countries we consider we have a mix of different vaccination timings, so that our observations appear to be valid under different scenarios. We leave for future work a further analysis where vaccination timing is taken into account.

## Supplementary Information

Supplementary Information.

## Data Availability

The data presented in this paper (in aggregated form) and the programs used to process it are openly accessible at https://github.com/GCGImdea/coronasurveys/tree/master/papers/2022-omicron_efficacy_paper. The microdata of the CTIS survey from which the aggregated data was obtained cannot be shared, as per the Data Use Agreements signed with Facebook, Carnegie Mellon University (CMU) and the University of Maryland (UMD). The programs used in this study have been written in R, and use libraries *base*, *useful*, *dplyr*, *data.table*, *zoo*, *reshape2*, *randomForest*, *nnet*, *parallel*, *ggrepel*, *caret*, and *tidyverse*. For completeness we have included a folder *pipeline* that contains the scripts that are used to process the microdata (as presented in eTable 11).
