# Supplementary material for: Using survey data to estimate the impact of the omicron variant on vaccine efficacy against COVID-19 infection
Source: Sci Rep. 2023 Jan 17;13:900. doi: 10.1038/s41598-023-27951-3 (PMC9844193; doi:10.1038/s41598-023-27951-3)
Supplement: Supplementary file 1 — Supplementary Information. [file 41598_2023_27951_MOESM1_ESM.pdf]

Using Survey Data to Estimate the Impact of the Omicron Variant on Vaccine Efficacy against COVID-19 Infection (Supplementary Information)

Jesús Rufino, Carlos Baquero, Davide Frey, Christin A. Glorioso, Antonio Ortega, Nina Reščič, Julian Charles Roberts, Rosa E. Lillo, Raquel Menezes, Jaya Prakash Champati, and Antonio Fernández Anta

A Curating of Responses

In this work, we use the responses to the UMD Global CTIS, to which we have access by agreement with UMD and Facebook (see Section 2.4). We first curate the data by removing abnormal responses, following the approach proposed by Alvarez et al. [29]: We remove responses that declare to have all symptoms or that declare unusual values (greater than 100) in the quantitative questions of the survey (e.g., days of symptom duration, number of symptomatic contacts, number of people staying at the same place, etc.).

B Machine Learning Classifier: Random Forest

B.1 Ground-truth Set

After curating the responses, the next task we face is determining whether they correspond to active cases of COVID-19. This is somewhat direct for the subset of responses that respond affirmatively to the survey question "B7: Have you been tested for COVID-19 in the past 14 days?" and then respond positively or negatively to the survey question "B8a: Did your most recent test find that you had COVID-19?" [28]. For this work, we assume that a participant responding affirmatively to both questions is an active case of COVID-19 (i.e., it is a *positive* case). Similarly, a participant responding affirmatively to Question B7 and negatively to Question B8a is assumed not infected with COVID-19 (i.e., *negative*). This set of classified responses constitute a *ground-truth set*, for which infection status (positive or negative) is available.

Unfortunately, this ground-truth set cannot be used directly to estimate the prevalence of COVID-19 in the overall population, because the set is usually very small and is not produced via uniform random sampling: People who have reason to believe they may be infected are more likely to be tested and therefore the ratio of positives among those tested in the latest 14 days (i.e., the *testing positive rate*, abbreviated TPR) is higher than the actual prevalence.

B.2 Creating the Machine Learning Classifier: Random Forest

Each response to the survey includes a large number of questions (obviously, not all participants answer all questions). For training and inference of the Random Forest classifier, we use only questions with answers holding discrete values. From these we remove questions B7 and B8, which are only used to create the ground-truth set, as well as related questions, such as "B0: As far as you know, have you ever had coronavirus (COVID-19)?" and "B15: Do any of the following reasons describe why you were tested for COVID-19 in the past 14 days?". Finally, we do not use the questions related to vaccination, since we do not want them to influence the classification. The set of questions used can be found in Appendix D. The answers to this set of questions are "dummified" before they are used, i.e., a question with k possible answers is replaced by k binary attributes. The Random Forest model is generated with the randomForest function in R. No hyperparameter tuning is done, and the standard options of the function are used, with the exception of limiting the model to 100 trees to reduce the training time.

Observe that the questions in Appendix D include all symptoms, but also have many more questions, including behavioral or demographic aspects. Additionally, the Random Forest classifier can give different weights to different symptoms, while previously proposed symptom based criteria are based on determining only whether a symptom is present or not. Thus, overall the Random Forest classifier is much more versatile than the symptom-based criteria described in the previous section. Additionally, there are other aspects that make the Random Forest classifier(s) more adaptive:

- Firstly, we create different models for different countries. It is expected that different countries will have local characteristics, thus training a different classifier for each country can capture them.
- Secondly, we create not one but several models per country: one for each 3-month period. This allows the model to capture and adapt to aspects that change over time, like the level of vaccination, the surge of new variants, or the stringency of measures imposed.

B.3 Evaluating the Classifiers

In order to verify whether the Random Forest classifier provides better proxy estimates than the symptoms-based classifiers, we selected a set of countries and tested the performance of each classifier in the last two quarters of 2021. To this end, we randomly divided the ground-truth set into a training and a testing set, with 70% and 30% of the responses of the ground-truth set in each subset, respectively. eTable 9 shows the results for three countries that have detected Omicron in December for the periods of July-September 2021 (2021-Q3) and of October-December 2021 (2021-Q4). The classification performance metrics used are:

- Accuracy: Ratio of cases correctly classified over the size of the test set.
- Sensitivity / recall: Ratio of cases correctly classified as positive over the number of positive cases.
- Specificity: Ratio of cases correctly classified as negative over the number of negative cases.
- F-score: Harmonic mean of precision and recall, where the precision is the ratio of cases correctly classified as positive over the number of all cases classified as positive.

As can be seen in eTable 9, Random Forest almost always shows the highest performance (marked in bold) among the classification methods used.

As another test, we then selected a set of countries that includes South Africa, along with the 20 countries that have the largest number of available responses in the UMD Global CTIS dataset. For each of these countries, the first two columns of eTable 10 show the official Test Positivity Rates obtained via *Our World In Data* [32, 36] (OWID TPR) and the corresponding survey-based estimate from the UMD Global CTIS dataset (CTIS TPR). The remaining columns show the Pearson correlation coefficient between the time series of Confirmed active cases (computed based on data from Johns Hopkins University [38] as described by Alvarez et al. [29]) and that of each of the candidate proxies in the period June 18th, 2021 (start of the first period considered in [16]), to December 31st, 2021. All time series have one value per day, which is the average of the latest 14 days.

We can make two observations from eTable 10. First, among all candidate proxies considered, Random Forest achieves at least 0.9 correlation for the largest number of countries. Second, 17 out of the 21 countries exhibit low TPR (≤ 0.1) values in at least one of the first two columns (either official or survey-based TPR), and 11 out of the 21 exhibit low values in both columns, with 7 having values no higher than 0.05 (the WHO considers countries to have the epidemic under control when their TPR is below 0.05 [34]). This suggests that such countries keep the case count under control and report more accurate official data on confirmed cases. We can thus interpret the higher correlation between the Random Forest proxy and the Confirmed time series for the countries with low TPR as a sign that this proxy constitutes the most promising option among the five proxies considered, and thus will also be more accurate for countries for which the official data will be less reliable.

C List of Symptoms

In the UMD Global CTIS the following question is asked: "B1 In the last 24 hours, have you had any of the following?" [28]. The following is the list of possible answers (non exclusive):

- Fever (B1_1).
- Cough (B1_2).
- Difficulty breathing (B1_3).
- Fatigue (B1_4).
- Stuffy or runny nose (B1_5).
- Aches or muscle pain (B1_6).
- Sore throat (B1_7).
- Chest pain (B1_8).
- Nausea (B1_9).
- Loss of smell or taste (B1_10).
- Headache (B1_12).
- Chills (B1_13).

D Questions Used for the Machine Learning Model

The following is the list of survey questions whose answers are used to create the Random Forest models, and to classify with them the responses: B1_1, B1_2, B1_3, B1_4, B1_5, B1_6, B1_7, B1_8, B1_9, B1_10, B1_11, B1_12, B1_13, B1_14, B1b_x1, B1b_x2, B1b_x3, B1b_x4, B1b_x5, B1b_x6, B1b_x7, B1b_x8, B1b_x9, B1b_x10, B1b_x11, B1b_x12, B1b_x13, B1b_x14, B3, B5, B6, B9, B10, B11, B12_1, B12_2, B12_3, B12_4, B12_5, B12_6, B13_1, B13_2, B13_3, B13_4, B13_5, B13_6, B13_7, B14_1, B14_2, B14_3, B14_4, B14_5, C0_1, C0_2, C0_3, C0_4, C0_5, C0_6, C1_m, C2, C3, C5, C6, C7, C8, C9, C9a, C12, C13_1, C13_2, C13_3, C13_4, C13_5, C13_6, C14, D1, D2, D3, D4, D5, D6_1, D6_2, D6_3, D7, D8, D9, D10, E2, E3, E4, E7, H1, H2, H3.

The questions removed are B0, B7, B8, B15, and all the questions related to vaccination (V-questions).

E Vaccination in South Africa

eFigure 1 shows an area plot, estimated from the UMD Global CTIS data, of the proportion of vaccinated with 1 dose, Vaccinated with 2 doses, and Unvaccinated from June 18th until December 31st, 2021. As can be seen, the ratio of the population vaccinated is low at the beginning of this interval, especially with two doses. Then, we can see a high increase in Vaccinated between July and October. We point out that in each time point of this plot the proportions are provided by a different set of surveys respondents, and it still closely captures the increase of vaccination.

eTable 1 shows the distribution of doses used and population vaccinated with the two types of vaccines delivered in South Africa: Johnson&Johnson and Pfizer/BioNTech. Some columns are inferred from the available data: total doses, people vaccinated, and people fully vaccinated. The dates shown are the closest available to the start and end of the intervals considered. This data has been obtained from Our World in Data [36]. In the same table, the rightmost columns present the percentage of responses to the UMD CTIS survey that report having received one or two doses of vaccination. As can be seen, these percentages are higher than the actual values (roughly for times higher in all dates for two doses) which hints that the respondents to the UMD CTIS survey are not a uniform sample of the population of South Africa.

F Countries with Omicron Prevalence

eTable 3 shows basic official vaccination data on December 31st, 2021, of these countries. eTable 4 shows the vaccine types delivered in these countries by the end of 2021. This data has been obtained from Our World in Data [32, 35, 36].

Tables 2 and 3 show the COVID-19 prevalence and the vaccine efficacy in October and December in the countries with presence of Omicron as defined in Section 2.3.2. When data is insufficient to meet the defined selection criteria, it is omitted and replaced by "–". Both tables are presented alphabetically by country name and also share a column depicting the most recent data on Omicron prevalence among all virus samples.

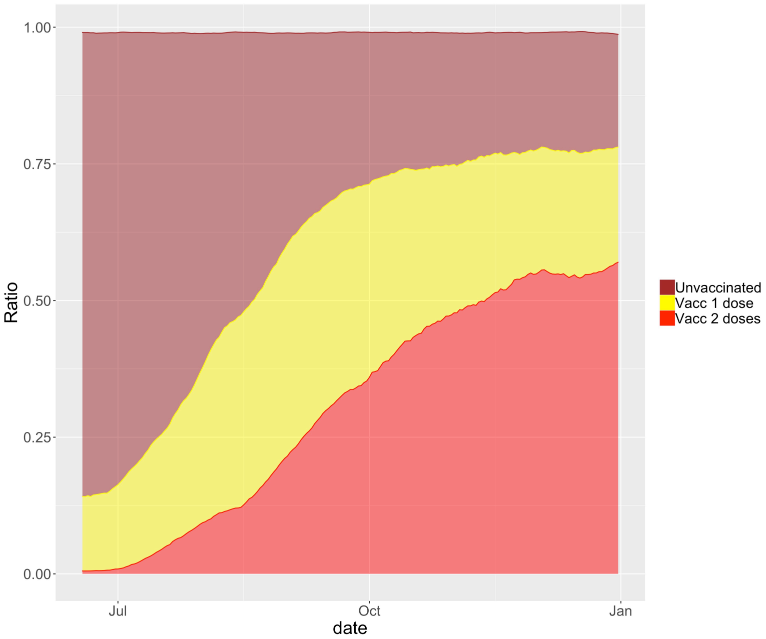

eFigure 1: Evolution of the vaccination in South Africa as ratio of the population, estimated from the UMD Global CTIS data. A small fraction of responses that declared being vaccinated without reporting the number of doses are not presented for clarity. The values are from June 18th to December 31st, 2021, smoothed with a rolling average of 14 days.

Date	Total doses	Doses J&J	Doses P/BNT	% pop fully vaccinated	% pop 1D			% pop 2D	% pop CTIS	
					J&J	P/BNT	Sum	P/BNT	1D	2D
2021/05/18	519139	479768	39371	0.81	0.81	0.06	0.87	0.00	5.57	0.21
2021/07/08	4017442	875575	3141867	2.14	1.49	4.07	5.56	0.64	18.14	1.99
2021/07/19	5095013	969525	4125488	2.88	1.65	4.58	6.23	1.22	21.49	5.16
2021/08/10	8811608	1903359	6908249	6.35	3.25	5.59	8.84	3.10	33.83	11.54
2021/09/07	13892301	3077591	10814710	11.52	5.25	5.91	11.16	6.27	38.83	25.15
2021/12/10	27043034	6631625	20411409	26.18	11.32	5.14	16.46	14.85	22.57	54.75
2022/01/01	27966664	6848461	21118203	27.10	11.69	5.24	16.93	15.40	21.12	57.37

eTable 1: Vaccine doses per manufacturer and percentage of the population vaccinated with each type in South Africa [36]. A person is fully vaccinated if it received one dose of Johnson&Johnson or two doses of Pfizer/BioNTech. The rightmost columns show the percentage of the UMD CTIS survey responses that declare having received one and two doses of vaccination, respectively. Abbreviations: Johnson&Johnson as J&J and Pfizer/BioNTech as P/BNT.

Date	% Delta	% Omicron	# samples
2021-06-14	45.23	0.00	1101
2021-06-28	78.09	0.00	1661
2021-07-12	88.90	0.00	2226
2021-07-26	94.30	0.00	1667
2021-08-09	95.19	0.00	1601
2021-08-23	97.58	0.00	1242
2021-09-06	97.01	0.00	1269
2021-09-20	95.77	0.00	923
2021-10-04	93.57	0.00	513
2021-10-18	93.56	0.00	450
2021-11-01	95.67	0.48	208
2021-11-15	69.30	20.18	114
2021-11-29	13.08	85.00	780
2021-12-13	0.92	95.92	980
2021-12-27	0.00	93.85	65

eTable 2: Percentage of sequenced virus samples belonging to Delta and Omicron in South Africa from June 1st to December 31st of 2021. The third column presents the total number of samples reported on the corresponding date.

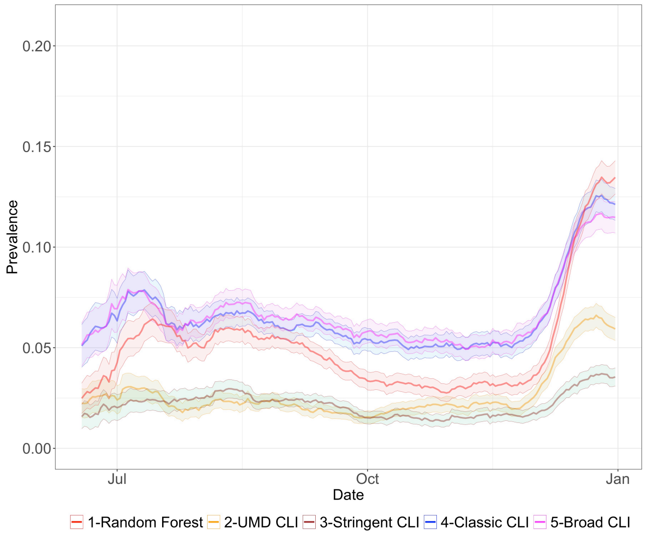

(a) Vaccinated

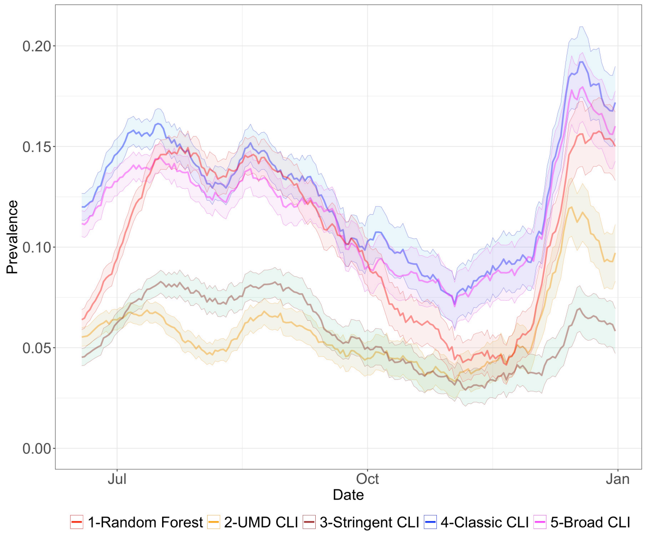

(b) Unvaccinated

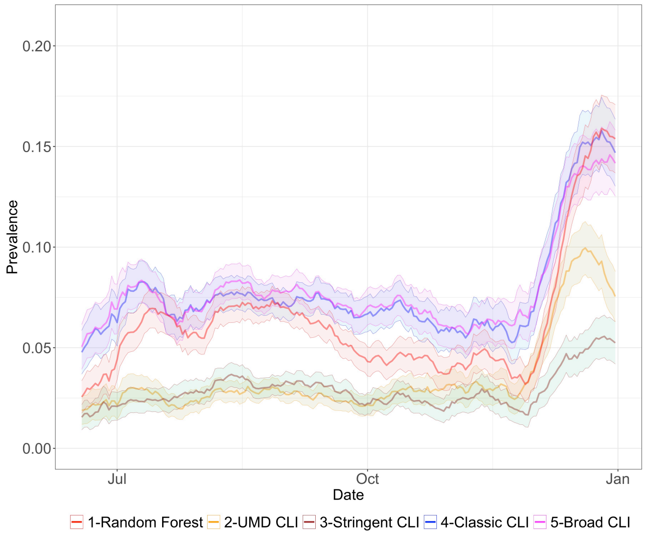

(c) Vaccinated with 1 dose

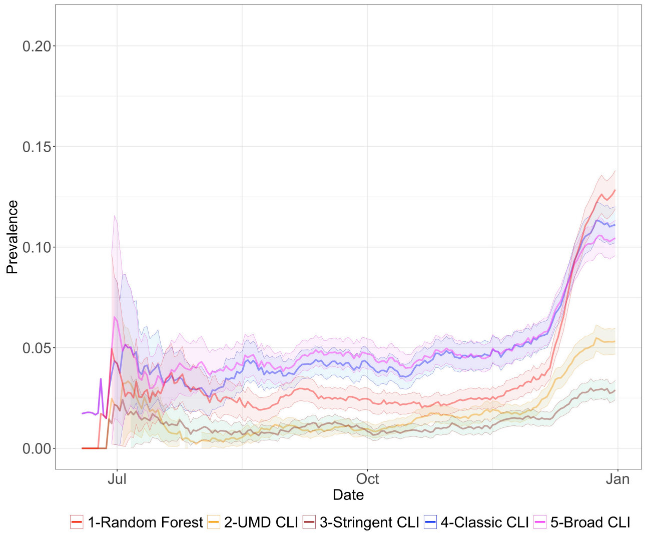

(d) Vaccinated with 2 doses

eFigure 2: Prevalence in South Africa among Vaccinated, Unvaccinated, Vaccinated with 1 dose, and Vaccinated with 2 doses, with different proxies.

Country	% doses/pop	% pop vacc	% pop fully vacc	% pop booster	Vacc start date
Argentina	167.98	83.76	71.61	12.22	2020-12-29
Belgium	186.28	76.65	75.70	37.59	2020-12-28
Brazil	154.81	77.66	67.03	12.42	2021-01-17
Colombia	126.19	74.81	55.25	6.49	2021-02-17
Denmark	208.57	82.65	78.43	48.30	2021-02-05
France	183.78	78.61	73.48	33.28	2020-12-27
Germany	178.84	73.62	70.61	38.87	2020-12-27
India	103.98	60.69	43.29	0.00	2021-01-16
Italy	184.28	80.14	74.11	32.52	2020-12-27
Mexico	114.24	62.89	55.87	0.00	2020-12-24
Netherlands	162.18	77.54	71.18	18.50	2021-01-09
Norway	178.68	78.41	71.76	28.52	2020-12-08
Poland	124.32	57.34	55.68	18.16	2020-12-28
Portugal	190.72	91.47	89.53	29.44	2020-12-27
Romania	82.86	28.64	40.87	0.00	2020-12-27
Russia	100.31	50.60	45.76	5.06	2020-12-15
Slovakia	111.09	50.13	47.61	16.33	2021-01-11
South Africa	46.47	31.49	26.37	0.00	2021-02-18
Spain	178.69	84.85	81.01	29.40	2021-01-04
Sweden	172.96	76.14	72.68	0.00	2021-01-03
Switzerland	158.90	68.56	66.88	24.99	2020-12-21
Turkey	154.80	66.92	60.68	27.19	2021-01-14
United Kingdom	195.45	75.93	69.54	49.98	2021-01-10
Vietnam	153.75	79.00	69.71	0.00	2021-03-08

eTable 3: Information about vaccination on December 31st, 2021, in the countries with presence of Omicron (as defined in Section 2.3.2).

Country	Vaccine
Argentina	CanSino, Moderna, O/AZ, P/BNT, Sinopharm/Beijing, Sputnik V
Austria	J&J, Moderna, O/AZ, P/BNT
Australia	Moderna, O/AZ, P/BNT
Bangladesh	Moderna, O/AZ, P/BNT,Sinopharm/Beijing
Belgium	J&J, Moderna, O/AZ, P/BNT
Brazil	J&J, P/BNT, O/AZ, Sinovac
Bulgaria	J&J, O/AZ, Moderna, P/BNT
Canada	J&J, Moderna, O/AZ, P/BNT
Chile	CanSino, O/AZ, P/BNT, Sinovac
Colombia	J&J, Moderna, O/AZ, P/BNT, Sinovac
Czechia	J&J, Moderna, O/AZ, P/BNT, Sinovac
Denmark	J&J, Moderna, P/BNT
Ecuador	CanSino, O/AZ, P/BNT, Sinovac
France	J&J, Moderna, O/AZ, P/BNT
Germany	J&J, Moderna, O/AZ, P/BNT
Greece	J&J, Moderna, O/AZ, P/BNT
Hungary	J&J, Moderna, O/AZ, P/BNT, Sinopharm/Beijing, Sputnik V
India	Covaxin, O/AZ, Sputnik V
Indonesia	J&J, Moderna, Novavax, O/AZ, P/BNT, Sinopharm/Beijing, Sinovac
Israel	Moderna, P/BNT
Italy	J&J, Moderna, O/AZ, P/BNT
Japan	Moderna, O/AZ, P/BNT
Malaysia	CanSino, O/AZ, P/BNT, Sinopharm/Beijing, Sinovac
Mexico	CanSino, J&J, Moderna, O/AZ, P/BNT, Sinovac, Sputnik V
Netherlands	J&J, Moderna, O/AZ, P/BNT
New Zealand	O/AZ, P/BNT
Nigeria	O/AZ
Norway	Moderna, P/BNT
Peru	O/AZ, P/BNT, Sinopharm/Beijing
Philippines	J&J, Moderna, O/AZ, P/BNT, Sinopharm/Beijing, Sinovac, Sputnik Light, Sputnik V
Poland	J&J, Moderna, O/AZ, P/BNT
Portugal	Covaxin, J&J, Moderna, Novavax, O/AZ, P/BNT, Sinopharm/Beijing, Sinovac
Romania	J&J, Moderna, O/AZ, P/BNT
Russia	Sputnik V, EpiVacCorona
Slovakia	J&J, Moderna, O/AZ, P/BNT, Sputnik V
South Africa	J&J, P/BNT
South Korea	J&J, Moderna, O/AZ, P/BNT
Spain	J&J, Moderna, O/AZ, P/BNT
Sweden	Moderna, O/AZ, P/BNT
Switzerland	J&J, Moderna, P/BNT
Taiwan	Medigen, Moderna, O/AZ, P/BNT
Thailand	Moderna, O/AZ, P/BNT, Sinopharm/Beijing, Sinovac
Turkey	P/BNT, Sinovac
Ukraine	J&J, Moderna, O/AZ, P/BNT, Sinovac
United Kingdom	Moderna, O/AZ, P/BNT
Vietnam	Abdala, Moderna, O/AZ, P/BNT, Sinopharm/Beijing, Sputnik V

eTable 4: Manufacturers of the vaccines delivered in the countries with presence of Omicron by December 31st, 2021 [36]. Abbreviations: Johnson&Johnson as J&J, Oxford/AstraZeneca as O/AZ, and Pfizer/BioNTech as P/BNT.

Country	Total Oct	Total Dec	Unvac Oct	Unvac Dec	Vac Oct	Vac Dec	Vac 1D Oct	Vac 1D Dec	Vac 2D Oct	Vac 2D Dec
Argentina	44509	48807	3077	2778	40276	44590	3704	1884	36115	41783
Belgium	16448	18373	1687	1718	14266	16004	747	463	13327	15269
Brazil	198423	162402	9428	6552	183859	151114	38885	8680	142594	139517
Colombia	34859	33883	5437	2734	28457	30197	9979	7514	18034	22137
Denmark	19591	27284	917	1206	18279	25472	212	217	17781	24684
France	82767	111041	10234	11593	67393	95663	6369	4708	60218	89139
Germany	89348	110359	12601	11868	71980	95530	6655	5490	64611	88548
India	76675	68155	4076	2631	63803	60076	16798	7344	45967	51622
Italy	98712	112754	7023	6095	89120	103305	9066	5108	78852	96124
Mexico	139967	118861	12063	6472	119471	109330	35960	17776	82321	90162
Netherlands	27505	30803	3804	3380	23001	26621	2175	2025	20397	24087
Norway	16746	21862	935	1010	15536	20404	389	304	14980	19724
Poland	30295	38001	5318	6105	23924	30578	2327	2499	21236	27603
Portugal	22758	29352	1299	1368	21017	27340	3470	3172	17180	23631
Romania	45123	24638	11038	4917	32558	19022	4477	2451	27594	16192
Russia	35186	30037	12301	9001	21680	19884	2845	2819	18573	16779
Slovakia	9567	11323	1987	2208	7382	8841	306	487	6989	8215
South Africa	18308	19492	4149	4006	12805	14753	5009	4138	7624	10423
Spain	33455	51568	2035	2625	30652	47444	3814	3574	26453	43223
Sweden	53564	57823	3001	3200	49564	53544	699	443	48380	52348
Switzerland	14863	16755	2906	2617	11585	13742	886	676	10541	12824
Turkey	27159	22854	3238	2307	23033	19844	1473	729	21015	18561
United Kingdom	41812	47072	3080	3174	37421	42421	925	770	36109	41122
Vietnam	48955	39105	8043	1116	37073	36097	17325	3241	19233	32246

eTable 5: Number of survey responses used in each period from the countries with presence of Omicron (as defined in Section 2.3.2), for each level of vaccination.

Country	Pos Oct	Pos Dec	Unvac Oct	Unvac Dec	Vac Oct	Vac Dec	Vac 1D Oct	Vac 1D Dec	Vac 2D Oct	Vac 2D Dec
Argentina	715	1302	87	99	594	1143	102	90	484	1034
Belgium	364	912	69	130	274	751	25	31	248	713
Brazil	5111	4066	405	224	4486	3648	1334	355	3072	3194
Colombia	1013	1103	285	158	666	897	291	280	364	596
Denmark	232	1405	24	116	196	1256	5	16	186	1228
France	703	3452	149	596	486	2733	102	130	377	2566
Germany	619	2253	155	580	428	1616	52	149	373	1453
India	2899	2231	186	93	1629	1235	623	242	958	939
Italy	558	2610	120	329	394	2158	67	95	322	2035
Mexico	6881	4747	1201	485	5167	4047	2287	1038	2808	2956
Netherlands	487	1441	95	210	367	1179	60	106	299	1046
Norway	147	569	15	39	127	516	10	17	116	495
Poland	1039	2504	298	749	676	1614	90	173	572	1416
Portugal	170	821	17	55	142	742	28	98	112	632
Romania	2579	448	1109	175	1335	239	158	42	1158	186
Russia	1550	775	752	318	727	401	79	70	633	323
Slovakia	276	635	89	216	174	397	14	36	157	360
South Africa	695	2348	249	599	388	1672	214	564	167	1093
Spain	468	2776	65	186	375	2479	80	177	290	2277
Sweden	297	1037	48	103	234	899	8	16	225	878
Switzerland	170	639	61	175	102	445	10	21	90	418
Turkey	1479	1143	288	181	1125	897	136	57	962	818
United Kingdom	1321	2168	141	180	1124	1926	53	59	1060	1851
Vietnam	364	1271	58	35	251	1141	95	76	152	1043

eTable 6: Number of survey responses classified as positive by Random Forest in each period from the countries with presence of Omicron (as defined in Section 2.3.2), for each level of vaccination.

	Prevalence		Vaccination efficacy	
Vaccination status	October	December	October	December
Vaccinated 2 doses	0.02 [0.01,0.02]	0.03 [0.03,0.04]	0.53 [0.49,0.58]	0.45 [0.39,0.50]
Vaccinated	0.02 [0.01,0.03]	0.04 [0.03,0.04]	0.49 [0.45,0.52]	0.43 [0.37,0.48]
Vaccinated 1 dose	0.03 [0.02,0.04]	0.05 [0.04,0.06]	0.34 [0.22,0.45]	0.32 [0.23,0.41]
Unvaccinated	0.04 [0.03,0.05]	0.06 [0.05,0.07]	–	–

eTable 7: Prevalence of COVID-19 and vaccine efficacy (with 95% confidence interval) in the countries with presence of Omicron in the periods of October and December 2021.

Prevalence omicron vs	Correlation coefficient	P-value
Vaccination efficacy	-0.680301	0.000354
Vacc. efficacy 1 dose	-0.564977	0.035274
Vacc. efficacy 2 doses	-0.628936	0.001306

eTable 8: Correlation between prevalence of Omicron and vaccine efficacy in the countries with presence of Omicron.

Country	Quarter	Classifier	Accuracy	Sensitivity	Specificity	F-score
Argentina	2021-Q3	Random Forest	**0.85**	0.80	**0.86**	**0.61**
		UMD CLI	0.78	0.74	0.79	0.25
		Stringent CLI	0.82	**0.85**	0.82	0.44
		Classic CLI	0.81	0.67	0.83	0.48
		Broad CLI	0.80	0.64	0.82	0.45
Japan	2021-Q3	Random Forest	**0.95**	**0.81**	**0.96**	**0.51**
		UMD CLI	0.94	0.58	0.95	0.36
		Stringent CLI	**0.95**	0.77	0.95	0.39
		Classic CLI	0.93	0.44	**0.96**	0.42
		Broad CLI	0.91	0.29	0.95	0.29
South Africa	2021-Q3	Random Forest	**0.83**	0.81	**0.83**	**0.71**
		UMD CLI	0.71	0.70	0.72	0.34
		Stringent CLI	0.79	**0.87**	0.77	0.57
		Classic CLI	0.77	0.71	0.80	0.61
		Broad CLI	0.76	0.70	0.78	0.57
Argentina	2021-Q4	Random Forest	**0.90**	**0.71**	**0.91**	**0.51**
		UMD CLI	0.88	0.63	0.89	0.35
		Stringent CLI	0.88	0.70	0.89	0.37
		Classic CLI	0.86	0.48	**0.91**	0.44
		Broad CLI	0.86	0.47	0.90	0.42
Japan	2021-Q4	Random Forest	**0.97**	**0.69**	**0.97**	**0.31**
		UMD CLI	0.96	0.26	**0.97**	0.20
		Stringent CLI	**0.97**	0.59	**0.97**	0.30
		Classic CLI	0.94	0.18	**0.97**	0.22
		Broad CLI	0.93	0.11	**0.97**	0.14
South Africa	2021-Q4	Random Forest	**0.83**	0.69	**0.85**	**0.55**
		UMD CLI	0.79	0.63	0.81	0.35
		Stringent CLI	0.80	**0.74**	0.80	0.32
		Classic CLI	0.80	0.58	0.84	0.48
		Broad CLI	0.80	0.58	0.84	0.47

eTable 9: Performance for three different countries in two different 3-month periods (2021-Q3: July-September 2021 and 2021-Q4: October-December 2021) of the different classifiers in the ground-truth set, when randomly divided into training (70%) and testing (30%) subsets.

			Pearson correlation with Confirmed				
Country	OWID TPR	CTIS TPR	Random Forest	UMD CLI	Stringent CLI	Classic CLI	Broad CLI
Argentina	**0.09**	0.17	**0.95**	**0.97**	**0.96**	**0.92**	**0.91**
Australia	**0.01**	**0.02**	**0.93**	0.46	0.31	-0.10	0.03
Brazil	–	0.19	**0.98**	0.03	0.82	0.36	0.46
Canada	**0.03**	**0.04**	**0.94**	0.85	0.66	0.73	0.71
France	**0.03**	**0.05**	**0.92**	0.69	0.80	0.57	0.61
Germany	**0.09**	**0.01**	**0.96**	0.88	**0.91**	0.82	0.81
Hungary	**0.08**	0.16	**0.93**	0.85	**0.95**	0.82	0.79
India	**0.02**	0.16	0.31	-0.38	-0.31	-0.71	-0.37
Italy	**0.02**	**0.03**	**0.98**	0.86	0.85	0.71	0.72
Japan	**0.05**	**0.04**	**0.93**	**0.90**	0.84	-0.17	0.67
Mexico	0.27	0.22	**0.97**	**0.99**	**0.98**	**0.95**	**0.98**
Poland	**0.08**	0.16	**0.96**	0.82	**0.97**	0.80	0.80
Romania	**0.07**	**0.09**	**0.94**	**0.96**	**0.98**	**0.96**	**0.95**
Russia	**0.05**	0.14	0.38	0.34	0.37	0.41	0.33
South Africa	0.16	0.24	**0.93**	**0.92**	0.84	**0.97**	**0.98**
Spain	**0.07**	**0.09**	**0.93**	0.82	0.79	0.48	0.52
Sweden	**0.06**	**0.05**	**0.91**	0.83	0.74	0.71	0.67
Thailand	0.20	**0.07**	0.85	0.83	**0.92**	0.84	0.77
Ukraine	0.20	0.16	**0.97**	0.87	**0.95**	**0.91**	0.89
United Kingdom	**0.04**	**0.06**	0.84	0.70	0.52	0.59	0.60
Vietnam	**0.06**	**0.02**	0.83	0.79	0.79	0.74	0.78

eTable 10: Test-positivity rate (TPR) obtained from OWID and extracted from the UMD Global CTIS data for the 20 countries with largest survey data and South Africa. Values of at most 0.1 are shown in bold. The rest of columns show the Pearson correlation coefficient of each different proxy with the Confirmed time series. Correlation values of at least 0.9 are shown in bold. The time period used is Jun 18th, 2021 to Dec 31st, 2021. The estimates have been smoothed with a rolling average of 14 days.

Script name	Description
run_pipeline.sh	Processes the CTIS microdata to generate estimates aggregated daily.
dates2microdata.R	Separate the CTIS microdata (responses) into files by date and country.
microdata2total.R	Aggregate the responses of each country by quarter in different files.
total2dummies.R	Remove abnormal responses and "dummify" of the data columns (see Section B.2).
model_rf_generation.R	Train a random forest model as described in Section B.2.
model_rf_symp_generation.R	Train a random forest model but only for symptomatic responses.
model_Xgboost_generation.R	Train an Xgboost model.
model_Xgboost_symp_generation.R	Train an Xgboost model but only for symptomatic responses.
dummies2aggregates.R	Compute estimates of active cases using symptoms combinations and ML models, and aggregate the data per day.
run.sh	Processes the aggregated CTIS estimates to produce the tables and plots for this paper.
script-variants-monthly.R	Computation of Omicron presence since December 15th, 2021.
script-TPR.R	Generation of data for eTable 10.
script-country-plots-data-create.R	Generation of data for ZA plots.
script-country-plots.R	Generation of ZA plots.
script-vaccination-plot-ZA.R	Generation of the vaccination plots for ZA.
script-efficacy-ZA.R	Generation of efficacy tables for ZA.
script-efficacy-ZA-Gauteng.R	Generation of efficacy tables for Gauteng.
script-efficacy-data-create.R	Generation of efficacy data for world countries.
script-efficacy-plots.R	Generation of efficacy plots for world countries.
script-efficacy-tables.R	Generation of efficacy tables for world countries.

eTable 11: Scripts used to process the data in this study. run_pipeline.sh invokes a series of R scripts as presented to transform the CTIS microdata into estimates of active cases aggregated per day. run.sh invokes R scripts to process the aggregated estimates and other data to produce the tables and figures presented in the paper.
